# Tuning structural and magnetic properties of Fe oxide nanoparticles by specific hydrogenation treatments

**DOI:** 10.1038/s41598-020-74188-5

**Published:** 2020-10-14

**Authors:** S. G. Greculeasa, P. Palade, G. Schinteie, A. Leca, F. Dumitrache, I. Lungu, G. Prodan, A. Kuncser, V. Kuncser

**Affiliations:** 1 National Institute of Materials Physics, Atomistilor 405A, 077125 Magurele, Romania; 2 National Institute for Laser, Plasma and Radiation Physics, 077125 Magurele, Romania; 3 Ovidius University of Constanta, 124 Mamaia Avenue, 9005127 Constanţa, Romania

**Keywords:** Magnetic properties and materials, Nanoparticles, Structural properties, Synthesis and processing

## Abstract

Structural and magnetic properties of Fe oxide nanoparticles prepared by laser pyrolysis and annealed in high pressure hydrogen atmosphere were investigated. The annealing treatments were performed at 200 °C (sample A200C) and 300 °C (sample A300C). The as prepared sample, A, consists of nanoparticles with ~ 4 nm mean particle size and contains C (~ 11 at.%), Fe and O. The Fe/O ratio is between γ-Fe_2_O_3_ and Fe_3_O_4_ stoichiometric ratios. A change in the oxidation state, crystallinity and particle size is evidenced for the nanoparticles in sample A200C. The Fe oxide nanoparticles are completely reduced in sample A300C to α-Fe single phase. The blocking temperature increases from 106 K in A to 110 K in A200C and above room temperature in A300C, where strong inter-particle interactions are evidenced. Magnetic parameters, of interest for applications, have been considerably varied by the specific hydrogenation treatments, in direct connection to the induced specific changes of particle size, crystallinity and phase composition. For the A and A200C samples, a field cooling dependent unidirectional anisotropy was observed especially at low temperatures, supporting the presence of nanoparticles with core–shell-like structures. Surprisingly high M_S_ values, almost 50% higher than for bulk metallic Fe, were evidenced in sample A300C.

## Introduction

Fe-based nanoparticles (NPs) show remarkable interest in the scientific community for various applications such as biomedicine (hyperthermia^1^, targeted drug delivery^2^, computed tomography and magnetic resonance imaging contrast agents^3,4^), catalysis^5,6^, magnetic fluids^7^, gas sensors^8^, high-density magnetic storages^9^, water treatment and environment protection^10,11^. For many technological applications, the Fe-based NPs are required to fulfill challenging demands such as narrow size distribution, crystallinity and stability in air. Along with the synthesis procedure, the annealing treatments offer a valuable tool to optimize Fe and Fe oxide based NPs with respect to specific applications. Simultaneous or post annealing treatments can influence morpho-structural^12–15^ and related magnetic parameters^16,17^.

Annealing treatments in hydrogen atmosphere performed at moderate temperatures were reported to influence considerably the phase composition and magnetic properties in Fe and Fe oxide based nanostructures. We previously used annealing treatments in hydrogen atmosphere to remove oxidation in various Fe based nanostructures^18–21^. Snovski^22^ used reduction of iron oxide and iron carbide NPs in H_2_ at 450 °C for 2.5 h to obtain a main α-Fe phase (91%). Kin^17^ improved the crystallinity and increased the saturation magnetization of Fe nanoparticles by treatment in H_2_ at 200 °C for 4 h. FeOOH nanorods were reduced in H_2_/Ar mixture and in H_2_, at temperatures between 300 and 500 °C^12^. The surprising effects of the annealing treatments in hydrogen atmosphere on the structural and magnetic properties of Fe-based NPs prepared by laser pyrolysis are studied in this report.

## Experimental details

Fe oxide nanoparticles were prepared by the laser induced pyrolysis technique, as detailed in^23^. In this study, the central gas nozzle has two concentric tubes with the central one used for reactive mixture of 2.5 mm internal diameter. The synthetic air was used as oxygen source, Fe(CO)_5_ vapors as iron sources and the pressure in the reaction chamber was 300 mbar. The focal spot size was kept constant at 2 mm. The iron oxide nanoparticles labeled here by sample A were prepared at a laser power of 45 W, while the temperature was 515 °C. The ethane flow (100 sccm) has a double role: of carrier gas for Fe(CO)_5_ vapors and of sensitizer at CO_2_ laser wave-length. The synthetic air flow was also 100 sccm. An Ar flow of 2000 sccm was introduced in the external nozzle tube, while two equal Ar flows of 150 sccm each were introduced on sides in order to flush the ZnSe windows.

Annealing treatments in hydrogen atmosphere were performed at 200 °C (sample A200C) and 300 °C (sample A300C), in order to reduce oxidation. The heat treatment under hydrogen was performed using an apparatus based on stainless steel tubing. Prior to annealing, the tubes were degassed for 2 h at 300 °C under 10^–3^ mbar. The samples were annealed afterwards for 4 h at the above temperatures in flowing hydrogen gas (99.9999% purity, 100 ml/min flow rate).

The phase composition was further investigated by X-ray diffraction (XRD) using a Bruker D8 Advance diffractometer with Cu K_α_ radiation (wavelength 1.5406 Å) and a LiF crystal monochromator. Rietveld refinements of the XRD data were performed with the MAUD software^24^.

The morphology and structure of the as-synthesized NPs was observed by transmission electron microscopy (TEM) and selected area electron diffraction (SAED) analysis, using a Philips CM 120ST (120 kV) Electron Microscope. Additional High Resolution Transmission Electron Microscopy (HRTEM) images have been also obtained using a JEOL 2100 Electron Microscope. The elemental analysis was performed by energy-dispersive X-ray spectroscopy (EDX) attached to a scanning electron microscope (Philips XL30 CP) with an acceleration voltage of 15 kV.

The magnetic measurements were performed using a Superconducting Quantum Interference Device (SQUID) magnetometer (MPMS 7T from Quantum Design). Mössbauer spectroscopy (MS) measurements were recorded on a constant acceleration spectrometer, in transmission geometry and using a ^57^Co(Rh) source. The temperature dependent spectra were collected via a close cycle cryostat (Janis) and a couple of field dependent spectra were collected via a cryomagnet (ICEOxford Ltd.). The NORMOS computer program^25^ was used for the least-squares fitting of the Mössbauer spectra. The isomer shifts were reported relative to α-Fe at room temperature.

## Results and discussion

The as-synthesized nanoparticles were analysed by EDX in order to evaluate the elemental composition. The sample is homogeneous and contains 11(3) at.% C, 53(1) at.% O and 36(1) at.% Fe. The resulted Fe/O ratio is 0.68 close to the specific value (0.667) for maghemite (γ-Fe_2_O_3_).

**Figure 1:**
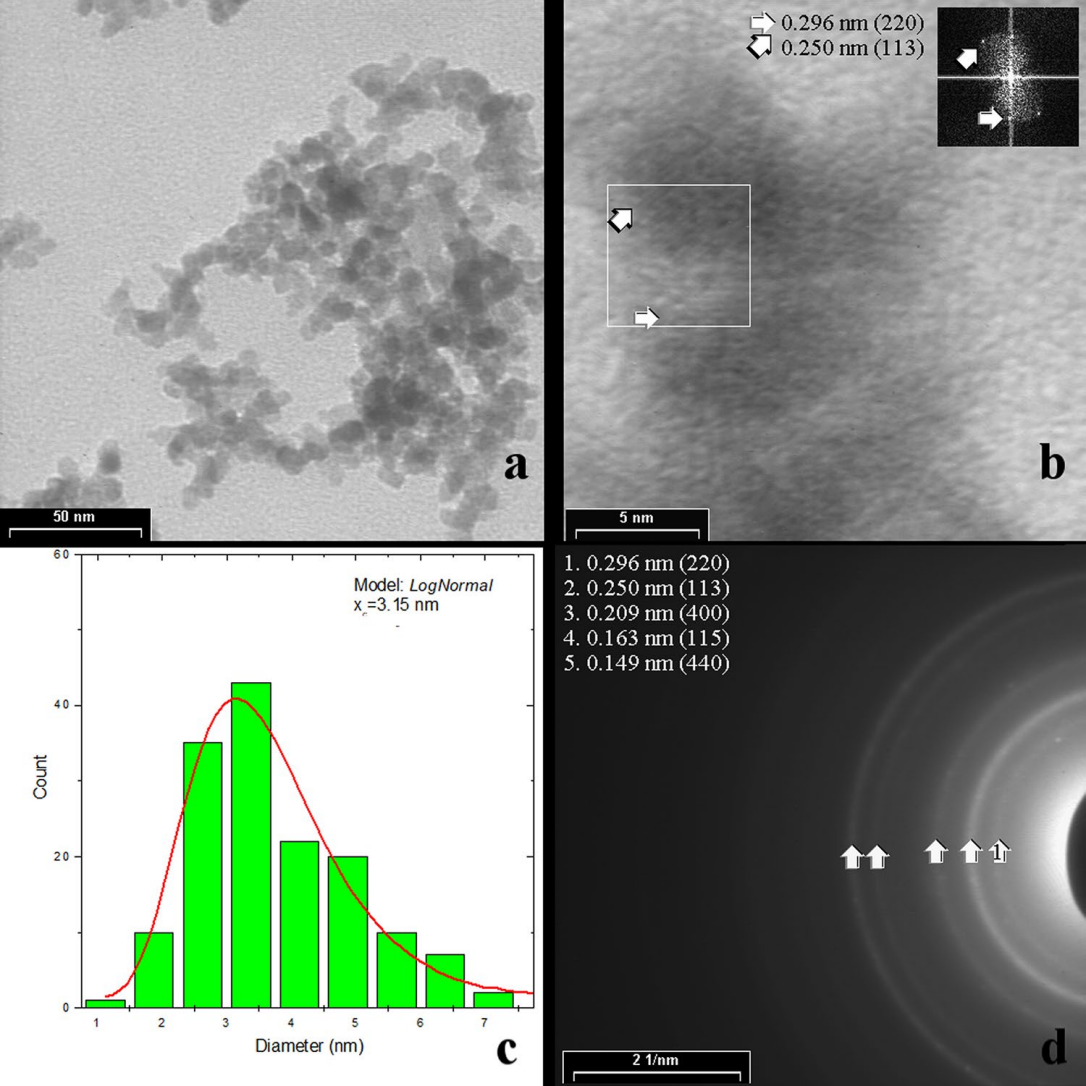
TEM analyses for sample A—as-synthesized nanoparticles: (**a**) TEM image at low resolution, (**b**) HRTEM image with an insertion containing the Fourier Transformed Image in order to evaluate inter-planar distances, (**c**) the particle size distribution fitted with Log normal function, and (**d**) a SAED image with identified inter-planar distances. Core–shell-like structures of NPs might be tentatively assumed according to the additional HRTEM data presented in the supplementary material.

TEM analysis and SAED images of sample A are presented in Fig. 1. The powder contains spherical shaped nanoparticles branched in a chain-like agglomeration (Fig. 1a). The particle size distribution is a mono-modal one and its fitting with Log-Normal Function provided a mean particle size of 3.15 nm (Fig. 1c). The HRTEM image (Fig. 1b) and the insertion with an image refining by Fourier Transformation revealed the internal crystalline structure of some NPs matching the (220) and (311) interplanar distances of γ-Fe_2_O_3_ crystalline phase. The SAED patterns (Fig. 1d) for as-synthesized powder exhibit diffuse rings that could be ascribed to Fe_3_O_4_ and/or γ-Fe_2_O_3_ phases (identified by the 2.52, 2.95, 1.61 and 1.48 Å reflections). There is no evidence in the SAED image for α-Fe or other Fe carbides around 2.01–2.05 Å distances.

**Figure 2:**
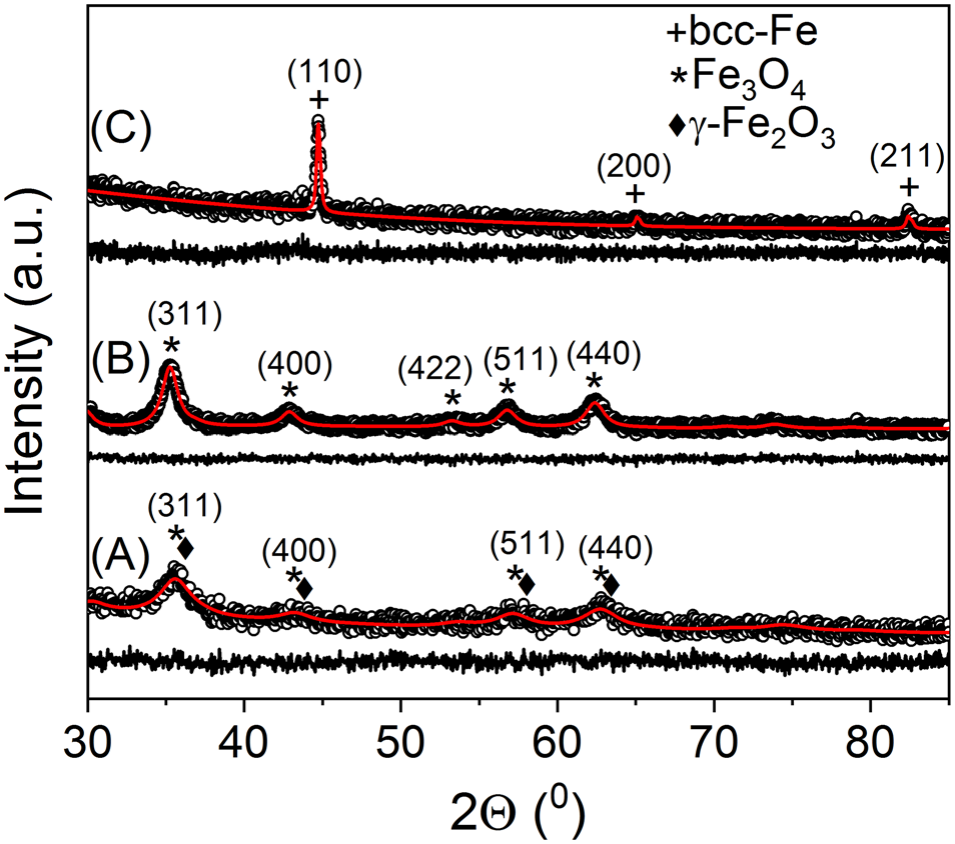
Rietveld refinements of the XRD data for samples: A (A), A200C (B), and A300C (C).

Rietveld refinements of the XRD data for samples A, A200C and A300C are given in Fig. 2. Table 1 shows the main crystallographic parameters resulted from Rietveld refinements as well as the reliability fit parameters. The broad and slightly asymmetric peaks of sample A indicate NPs with specific structure in-between of very distorted magnetite (Fe_3_O_4_) and maghemite (ICCD file 01-080-6402), with small crystallite size of 5.6 nm and high r.m.s. microstrain of 0.006%. Sample A200C contains NPs of only better formed magnetite (ICCD file 01-080-7683) due to the partial reduction of maghemite. No traces of metallic iron are observed. The crystallite size increases up to 10.3 nm and the microstrain decreases down to 0.003% compared with sample A. The lattice constant of the sample A200C (0.8406 nm) is roughly similar with one of sample A (0.8391 nm). The complete conversion of magnetite into metallic iron with bcc structure (ICCD file 04-007-9753) has been obtained in sample A300C. The crystallite size increases considerably up to 57.8 nm, whereas the microstrain diminished few times relative to the sample A200C.

**Table 1:** Crystallographic parameters resulted after Rietveld refinement of XRD data for samples: A, A200C, and A300C.

Sample	Lattice parameter (nm)	Crystallite size (nm)	R.M.S microstrain (%)	Fit reliability parameters
A	0.8391 (8)	5.6 (6)	0.006 (2)	GOF = 1.040 R_wp_ = 1.20% R_B_ = 0.96%
A200C	0.8406 (2)	10.3 (6)	0.003 (1)	GOF = 1.006 R_wp_ = 1.11% R_B_ = 0.91%
A300C	0.2865 (1)	57.8 (4)	0.0005 (3)	GOF = 1.045 R_wp_ = 5.84% R_B_ = 4.63%

In case of magnetic single-domain and non-interacting particles, the superparamagnetic relaxation is described by an Arrhenius-like Néel–Brown law^26–28^:

1 $$ \tau = \tau_{0} e^{{\left( {KV/k_{B} T} \right)}} $$

where *τ* is the Néel relaxation time, *τ*_0_ is the attempt time (characteristic of material and slightly dependent on temperature^29^) usually found in a wide range of 10^−9^–10^−12^ s, *k*_*B*_ is the Boltzmann constant, *K* is the magnetic anisotropy constant, *V* is the particle volume, *T* is the temperature.

Magnetic NPs are in a magnetic dynamic (superparamagnetic) regime above a temperature called blocking temperature (*T*_*B*_) whereas at temperatures lower than *T*_*B*_, they are in a magnetic frozen regime specific of a bulky-like material. *T*_*B*_ can be defined as the temperature for which the superparamagnetic relaxation time equates the measuring time window of the experimental method (*τ*_*M*_). It results:

2 $$ T_{B} = KV\left( {\ln \tau_{M} /\tau_{0} } \right)^{ - 1} /k_{B} $$

*τ*_*M*_ is specific to the employed experimental technique and therefore *T*_*B*_ is not uniquely defined. In magnetometry, *T*_*B*_ is investigated under the zero-field cooled—field cooled (ZFC–FC) protocol. In the ZFC sequence, the magnetization curve is obtained when the sample is cooled in the absence of a magnetic field and then the magnetization is measured at increasing temperature under a very small applied field, removing the degeneracy of the two minima of the magnetic anisotropy energy. In the FC sequence, the magnetization curve is obtained when the sample is initially cooled down in the same small applied magnetic field, which also remains applied during the measuring process at increasing temperature. The typical measuring time, *τ*_*M*_, for DC magnetometry is about 10 s and conventionally *T*_*B*_ is provided by the maximum of the ZFC curve.

**Figure 3:**
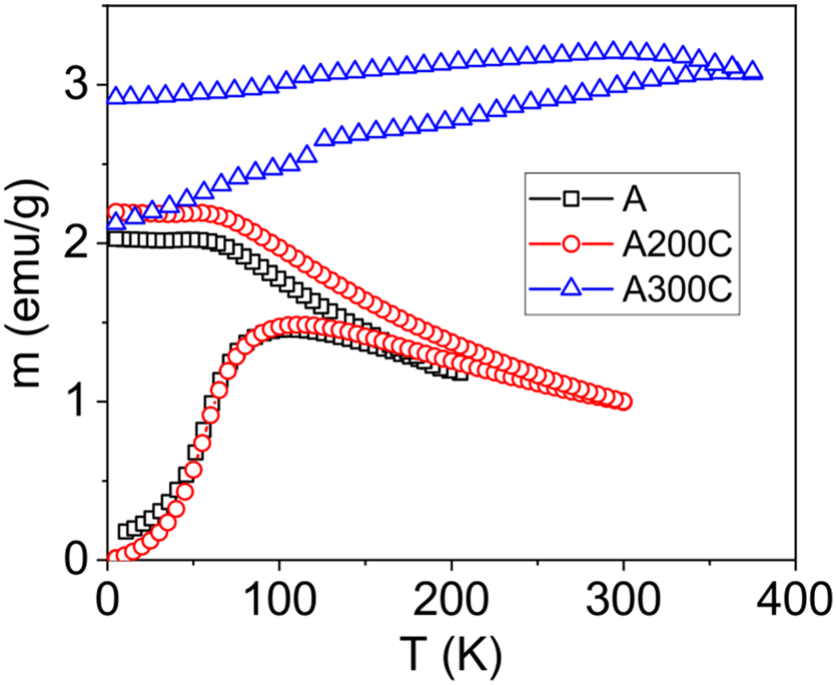
The ZFC–FC curves of the as prepared and annealed samples, A, A200C and A300C, in an applied field of 50 Oe.

The ZFC–FC curves of samples, A, A200C and A300C, measured in a field of 50 Oe, are presented in Fig. 3. Specific to NPs in sample A is *T*_*B*_ = 106(1) K corresponding to a magnetization of 1.46 emu/g. The ZFC curve for the sample A200C provides only an almost insignificant slightly increased *T*_*B*_ of 110(1) K, corresponding to a magnetization of 1.49 emu/g. However, the very close blocking temperatures associated to NPs in the two samples in conditions of a significant variation of the average particle size (about 5 and 10 nm, respectively) clearly indicate via relation (2) a significant difference of the corresponding anisotropy constants in the two samples (e.g. a few times lower *K* in sample A200C as in A) which was assumed to be related to a different oxidation state of Fe in the oxide NPs. To note the slight decrease of the ZFC curves at T > T_B_ for both samples A and A200C, providing evidence for either a very large size distribution of NPs and/or the presence of a fraction of interacting NPs. In case of sample A300C, the nanoparticle size increases about 5 times relative to sample A200C. According to relation (2), T_B_ should increase by 125 times in conditions of a same anisotropy constant of NPs in sample A200C and A300C. The experimental increase of T_B_ at only some 350 K in sample A300C is therefore in direct agreement with a decrease of the anisotropy constant of NPs in this sample by more than one order of magnitude relative to sample A200C. This can be simply explained by the phase composition of the involved NPs in sample A300C, dealing with single-phase metallic Fe, as revealed by XRD and also subsequently presented by MS results. However, the increasing trend of ZFC profile of sample A300C and the clear branching point at about 350 K indicate a low amount of superparamagnetic NPs above 350 K, the main signal corresponding rather to a long range magnetic structure specific to strongly interacting magnetic single-domain NPs.

**Figure 4:**
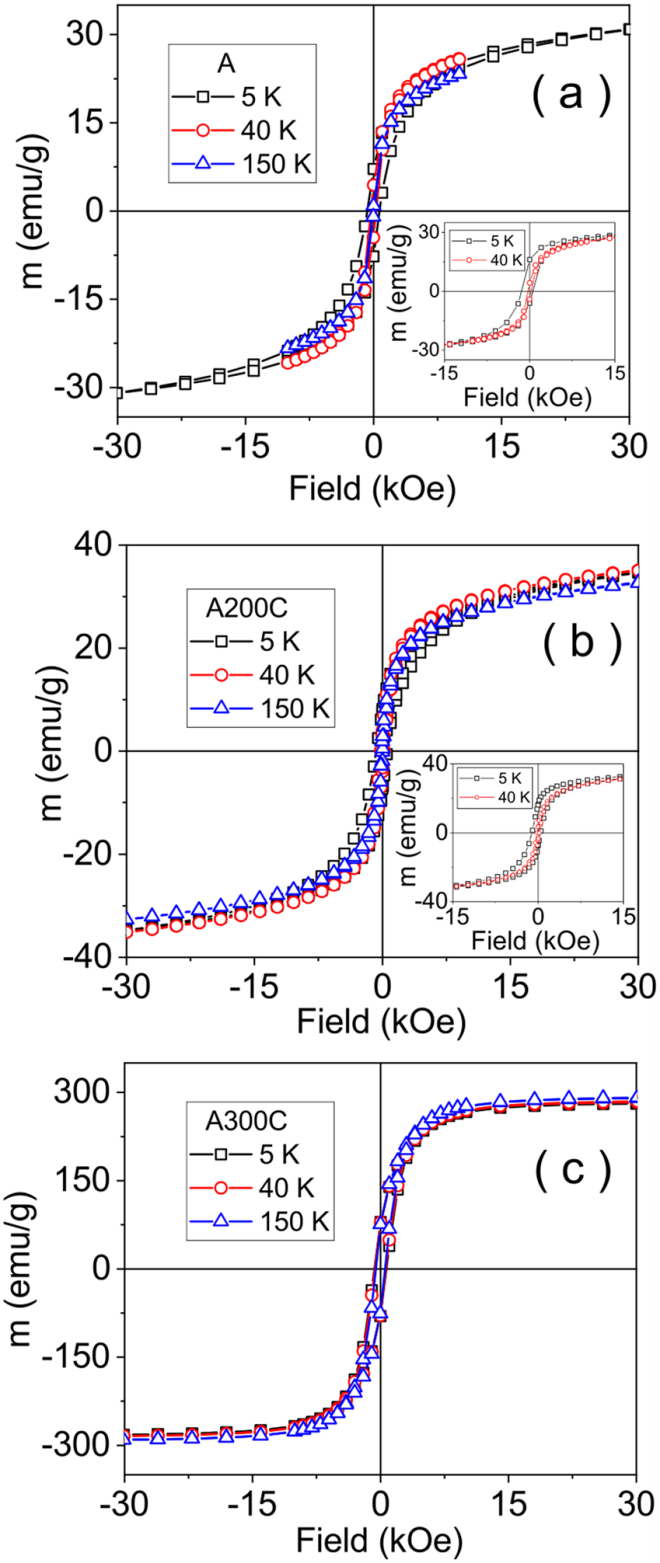
Hysteresis curves of the as prepared and annealed samples: A (**a**), A200C (**b**) and A300C (**c**). The inset of each figure shows the hysteresis curves collected after cooling the sample in a field of 50 Oe.

**Figure 5:**
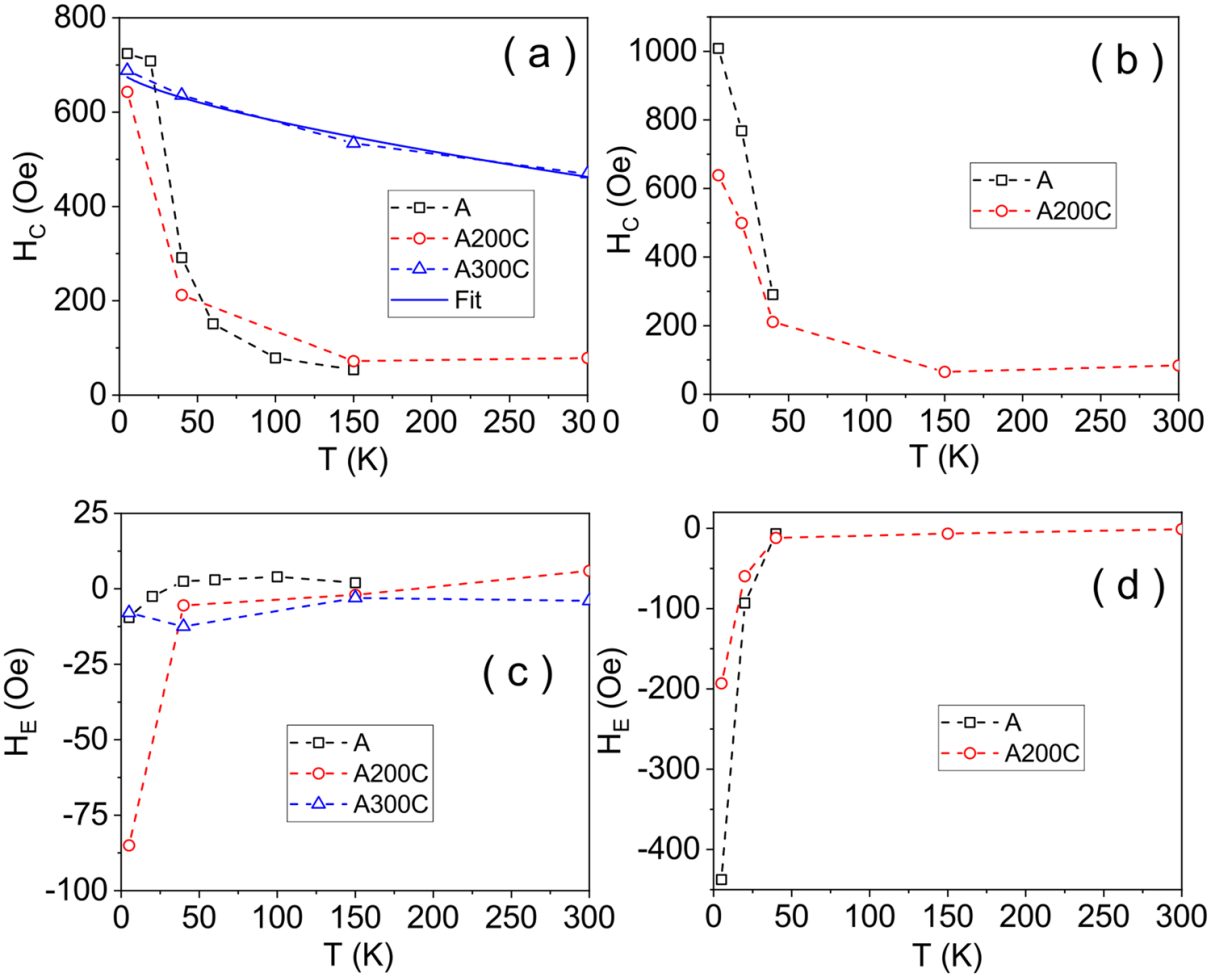
Temperature dependence of coercive field before (**a**) and after (**b**) in-field cooling procedure. Temperature dependence of exchange bias field before (**c**) and after (**d**) in-field cooling procedure.

The magnetic hysteresis curves of samples A, A200C and A300C, are shown in Fig. 4. The insets of Fig. 4 show the hysteresis curves of the mentioned samples collected after cooling the sample in an external magnetic field of 50 Oe. To note the specific negative shifts of the hysteresis loops in the case of samples A and A200C, better evidenced after cooling the samples in an applied field. This well-known behavior is due to the presence of unidirectional anisotropy in a nanometer size ferromagnetic-like phase interfaced to an antiferromagnetic-like phase^30–32^. Among the macroscopic effects of such interfacial interactions are: (1) the increased coercivity of the multi-phase system and (2) the above mentioned shift of the hysteresis loop, denoted as an exchange bias field. Figure 5 shows the evolution of both the coercive field (H_C_) and exchange bias field (H_E_) with temperature. The H_C_ values change considerably in the case of samples A and A200C, from > 700 Oe at low temperatures down to tens of Oe at room temperature (RT) recalling the typical dependence specific to superparamagnetic and non-interacting NPs^33^:

3 $$ H_{C} = H_{0} \left[ {1 - \left( {\frac{T}{{T_{B} }}} \right)^{1/2} } \right] $$

where *H*_0_ is the coercive field in the magnetically blocked regime, e.g. at 0 K. Low finite H_C_ values with an almost constant trend above *T*_*B*_ (< 150 K) give support for a small fraction of magnetically blocked (large size) NPs in these samples. For the sample A300C, H_C_ decreases slowly with temperature, remaining at a still very high value at temperatures close to 350 K, considered as the blocking temperature for the small amount of non-interacting Fe nanoparticles. The observed deviation from the specific dependence (3) gives support for strongly interacting NPs in this sample.

As above mentioned in the case of Fe oxide NPs, the evidenced negative *H*_*E*_ values at low temperature suggest the presence of interactions between antiferromagnetic or spin disordered oxides and ferrimagnetic oxide phases, most probably in a core–shell-like configuration (which is also probed by MS results and the micromagnetic computations presented in the supplementary material). As expected, the exchange bias interaction is stronger after cooling the sample in the presence of a magnetic field (Fig. 5d). As a result of the field cooling procedure, *H*_*E*_ increases more than double for sample A200C and one order of magnitude for sample A (these results will be also corroborated with the phase composition obtained from MS results in Figs. 8 and 9). The presence of the unidirectional anisotropy at low temperatures imposes also the strong increase of the coercivity in samples A and A200C, this additional effect being the reason for which the Eq. (3) is not valid for these samples too. It is to be noted here that the exchange bias field is cancelled out at a temperature of 50 K, also known as the blocking temperature of exchange bias^31^, being marked in the following as *T*_*EB*_. Hence, samples A and A200 are representative cases of nanoparticulate systems with *T*_*EB*_ < *T*_*B*_*.*

**Figure 6:**
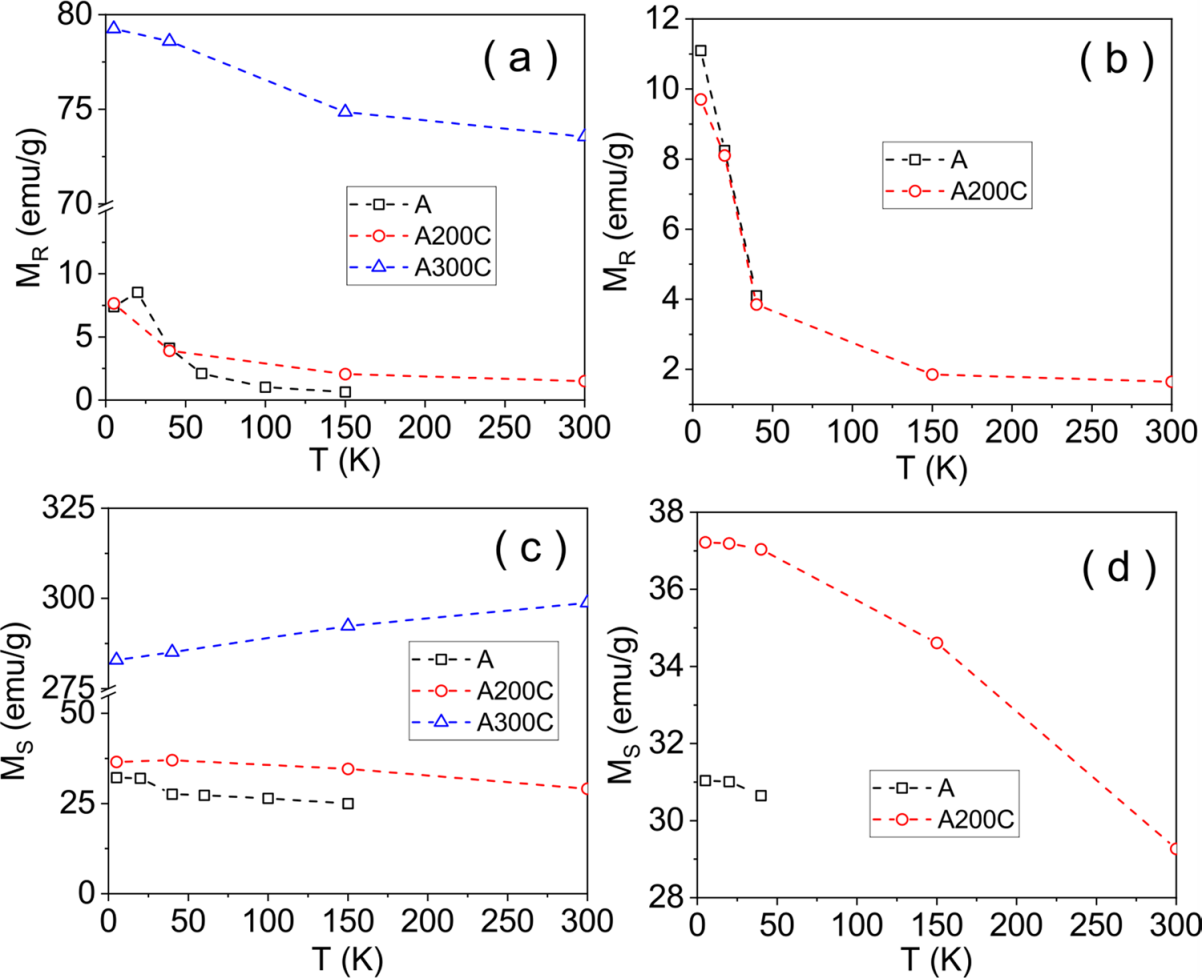
Temperature dependence of the remanent magnetization in the absence (**a**) and in the presence of field cooling (**b**). Temperature dependence of the saturation magnetization in the absence (**c**) and in the presence of field cooling (**d**).

The evolution of remanence (*M*_*R*_) and saturation (*M*_*S*_) magnetization with temperature is shown in Fig. 6. *M*_*R*_ values of samples A and A200C are roughly similar, with a slightly increased contribution after the field cooling procedure and a much slower decrease with temperature above T_EB_. Such specific behaviors of remanence provide evidence for its direct relation to the unidirectional anisotropy induced at the interface of the two distinct magnetic phases in the core–shell structure of oxide NPs. On the other hand, the much higher remanence of metallic Fe NPs in sample A300C has to be related only to the long range magnetic order inside the unidimensional chain-like organization of NPs, initially oriented along the saturation field^34,35^.

The *M*_*S*_ values are atypical for all samples. According to the EDX characterization, the relative content of C of 11% at. should lead to an increase of less than 5% in the saturation magnetization (in emu/g) if counting only the magnetic constituent. An unexpected *M*_*S*_ variation of Fe NPs, which decreases from 310 emu/g at 300 K to 290 emu/g at 10 K as well as the unexpected high *M*_*S*_ value at 300 K, i.e. some 50% higher than for bulk Fe, should be mentioned for sample A300C. We tentatively relate this behavior to local changes of the electronic configurations due to the random penetration of C atoms in the bcc structure of Fe, which might represent also the reason for the decreased anisotropy constant of NPs in sample A300C and for their magnetic single-domain structure. However new experimental and theoretical studies on such hydrogenated samples are required for a deeper understanding of their magnetic behavior.

On the other hand, the *M*_*S*_ values of the samples A and A200C are much reduced in comparison to specific values of the spontaneous magnetization of maghemite (about 75 emu/g) and magnetite (about 90 emu/g). For example, *M*_*S*_ for the magnetic component (maghemite-like) in sample A is about 32 emu/g at 10 K, i.e. only 43% from the spontaneous magnetization of a well formed maghemite, whereas *M*_*S*_ for the magnetic component (magnetite-like) in sample A200C is about 39 emu/g at 10 K, i.e. again only about 43% from the spontaneous magnetization of a well formed magnetite structure. Such huge discrepancies cannot be explained by a simple poor degree of crystallinity in the two samples, but rather via a core–shell structure of nanoparticles with a better formed ferrimagnetic core and a magnetic disordered shell (magnetic dead layer) with similar oxidation states as in the core. Assuming the typical maghemite magnetic structure in the NPs core and taking into account that only 43% of Fe ions are in the core of the 5 nm sized NPs in sample A, an average core size close to 3.5 nm and a surrounding magnetic dead layer close of 1.5 nm can be deduced. Similarly, in the case of sample A200C, NPs of average size of 10 nm will consist of a core with magnetite-like magnetic structure and average size close to 6.5 nm and a surrounding magnetic dead layer of about 3.5 nm.

**Figure 7:**
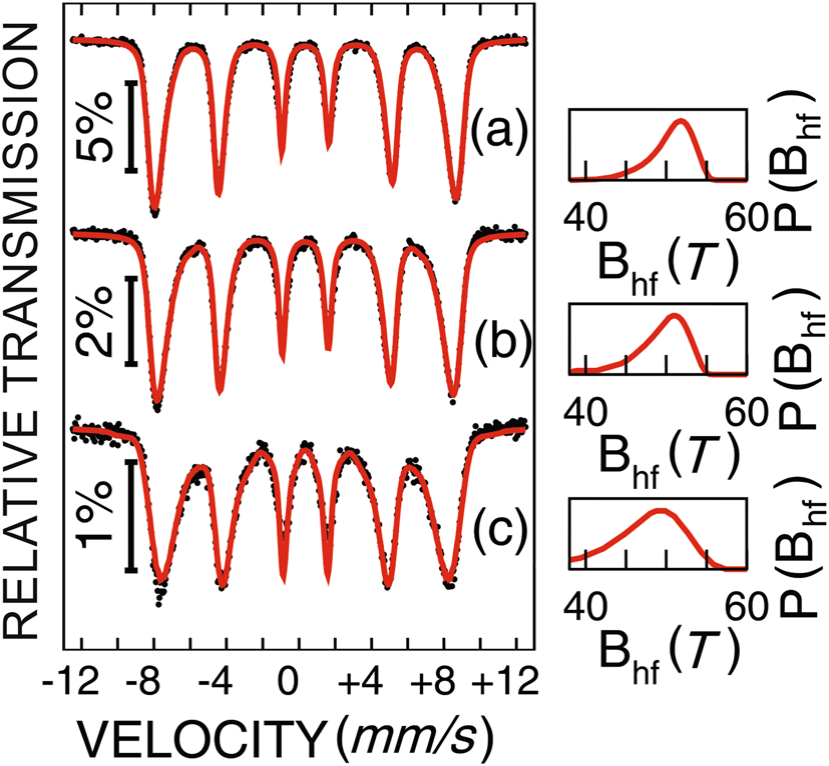
Mössbauer spectra of the as-prepared sample A, collected at low temperatures: 5 K (**a**), 30 K (**b**), and 60 K (**c**).

**Figure 8:**
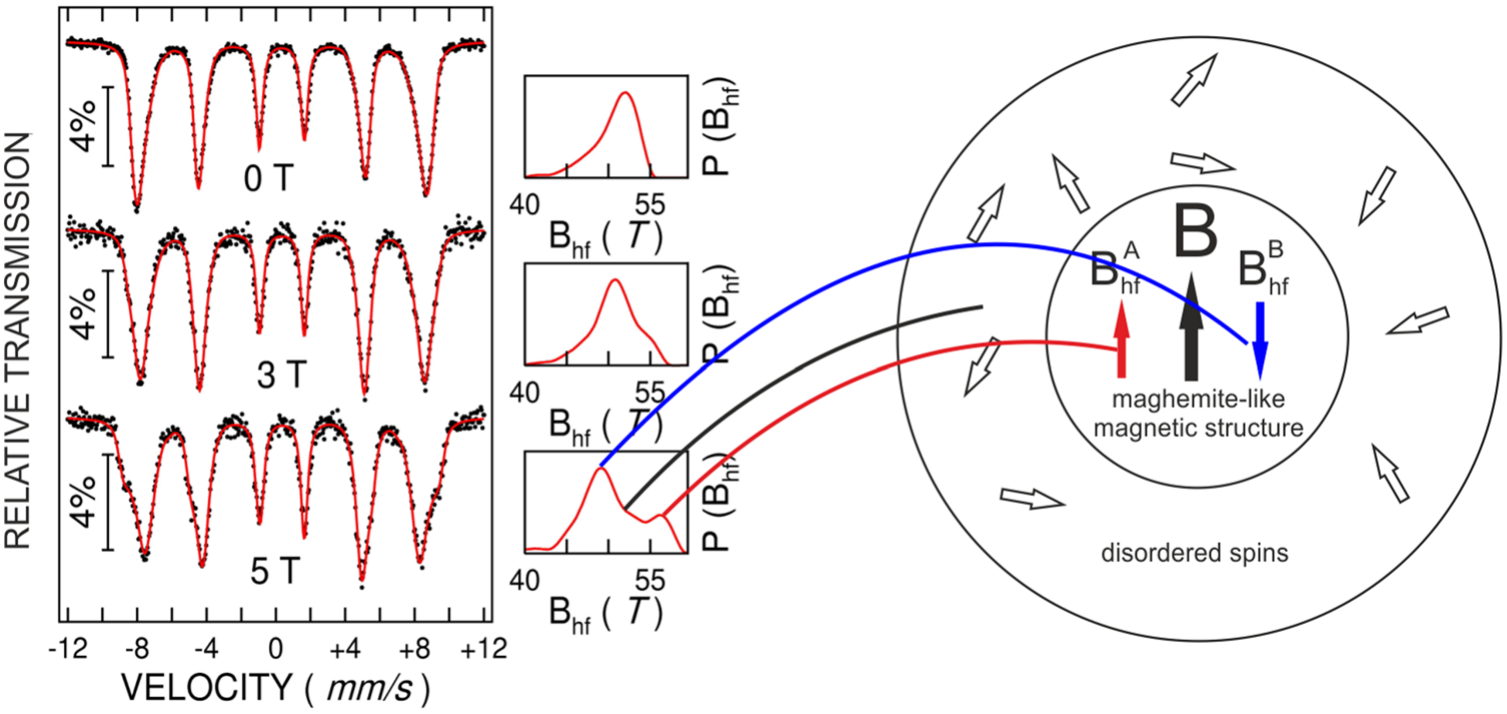
Mössbauer spectra of the as-prepared sample A, collected at 3 K without applied magnetic field (**a**), under 3 T applied magnetic field (**b**) and under 5 T applied magnetic field (**c**). A graphical representation of the spin structure specific to a magnetic NP with more ordered spins in the core and more disordered spins in the shell, in direct relation to the observed hyperfine magnetic field distributions, is shown in (**d**).

**Figure 9:**
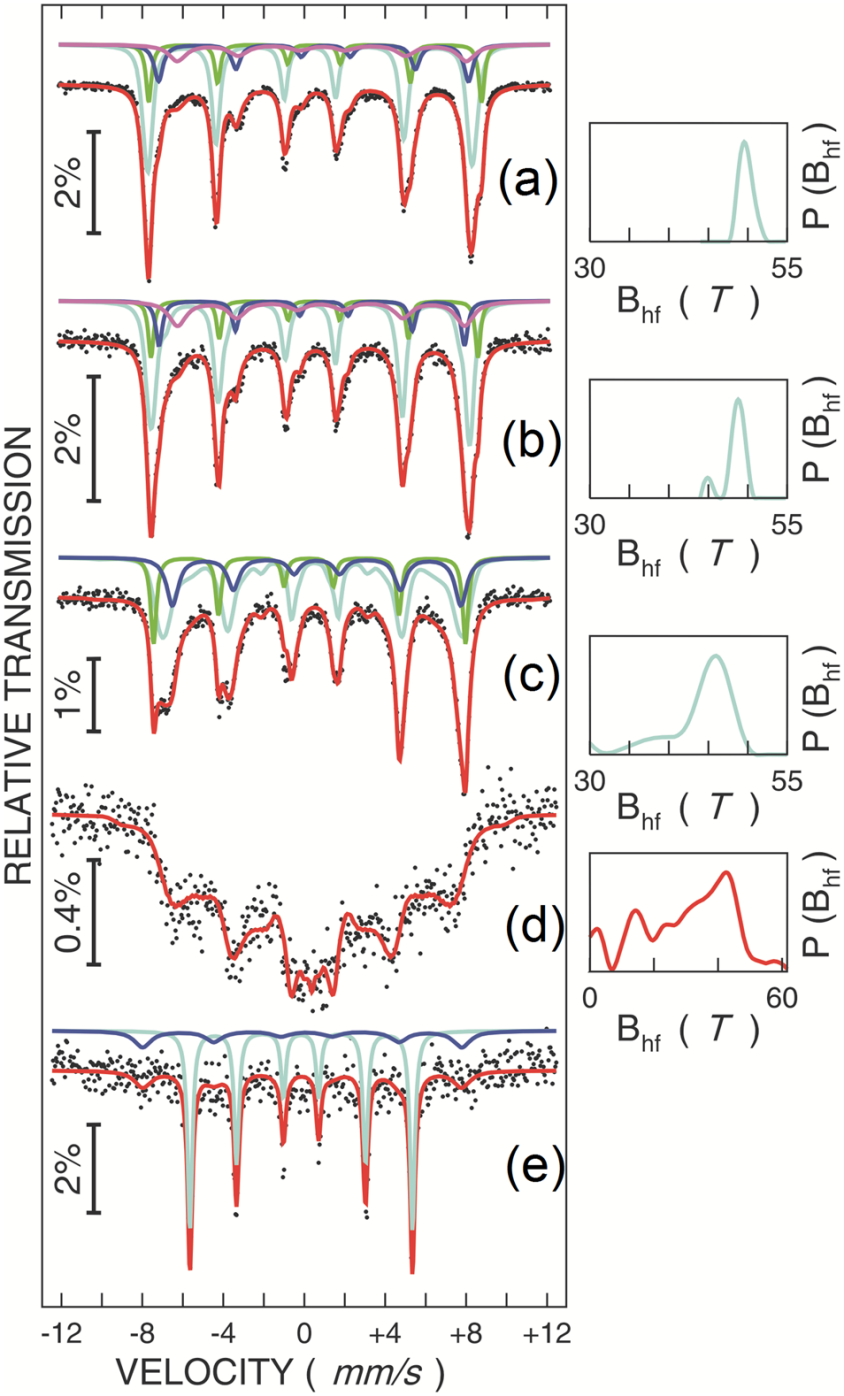
Mössbauer spectra of samples: A200C, collected at 5 K (**a**), 80 K (**b**), 160 K (**c**), room temperature (RT) (**d**); A300C, collected at 6 K (**e**). In the case of sample A200C, the probability distributions of hyperfine magnetic field are shown on the right side of the spectra. The unshaped broad distribution specific to the 300 K spectrum is due to magnetic relaxation effects.

The Fe phase composition, local structure and magnetic relaxation phenomena were investigated by temperature dependent ^57^Fe MS. The Mössbauer spectra of samples A, A200C and A300C, collected at different temperatures, are shown in Figs. 7, 8 and 9. The spectra of sample A, collected at low temperatures present a relatively broad sextet pattern and are fitted using the hyperfine magnetic field probability distribution method (Fig. 7).

On the other hand, the low temperature spectra of the annealed samples show rather narrow magnetic patterns and are fitted with either superposed hyperfine magnetic field distributions and discrete sextets (sample A200C) or only a discrete sextet component (sample 300C).

The Mössbauer spectra of the as prepared sample A with the hyperfine field distributions presented on the right side of Fig. 7 show at 5 K a most probable hyperfine magnetic field of more than 52 T, specific to mainly Fe^3+^ ions and suggesting the presence of distorted maghemite. The almost linear decrease of the most probable and average hyperfine magnetic fields with temperature is due to magnetic relaxation phenomena as will be subsequently discussed.

According to the saturation magnetization data, a magnetic dead layer (with random orientation of the Fe^3+^ spins) has to be considered at the surface of NPs in sample A, with a corresponding contribution included within the same overall hyperfine field distribution. A more direct proof for such a core–shell magnetic structure of NPs in sample A is provided by in field MS. Field dependent Mössbauer spectra collected at a temperature of 3 K are shown in Fig. 8 together with corresponding hyperfine magnetic field distributions (on the right side of each spectrum).

The Mössbauer spectrum collected in the cryomagnet at 3 K and in the absence of the applied magnetic field, was fitted according to the previous discussion via a unimodal distribution of hyperfine magnetic fields (Fig. 8a). If a magnetic field is applied perpendicular to the direction of the γ rays, the hyperfine field distribution initially starts to enlarge (Fig. 8b). Under a 5 T applied field, the hyperfine magnetic field distribution transforms into a bimodal one, with an intense local maximum at about 47 T and a less intense one at about 57 T (Fig. 8c). Such values correspond to average effective fields obtained by the superposition of the internal hyperfine magnetic field and the applied one. The 5 T applied magnetic field induces the almost complete reorientation of the net magnetic moments of the ferrimagnetic structure along the field direction, effect which is also clearly evidenced by the increased intensity of the second and fifth absorption lines of the sextet under increasing applied magnetic fields^28,36^). Hence, the two above mentioned effective fields have to correspond to Fe spins opposite to the external field (B_1_ = 47 T) and along the external field (B_2_ = 57 T), respectively. In both cases the effective field is obtained by subtracting/adding the extern field (5 T) to the hyperfine field (52 T). In the case of a defected spinel-like structure (cation deficient) as maghemite is^37^, the highest number of Fe^3+^ ions belong to octahedral-like B positions providing also the net magnetic moment of the compound which is oriented along the field. To note that the hyperfine magnetic field of mainly Fermi-contact origin is antiparallel to the magnetic moment of Fe. As a direct consequence, the hyperfine magnetic field associated to Fe^3+^ ions on such positions ($B_{hf}^{B}$ ) is antiparallel to the applied field, giving rise to the stronger component of the hyperfine field distribution centered on 47 T (Fig. 8d).

Concerning the distribution of the hyperfine magnetic field under a 5 T applied magnetic field, it can be also clearly observed a definite probability of effective fields centered on 52 T, providing evidence for a significant amount of randomly oriented magnetic moments/spins/ hyperfine fields associated to Fe^3+^ ions in the shell of the nanoparticle (a valid solution for the decomposition of the hyperfine magnetic field distribution by 3 components provides a median component of more than 50% contribution, in reasonable agreement with the magnetic measurements).

The spectra collected at temperatures up to room temperature on sample A200C (Fig. 9a–d), reveal relatively narrow magnetic patterns which evolve with temperature due to magnetic relaxation effects clearly evidenced by the broad collapsing magnetic pattern specific to the RT spectrum. The best fit of the 5 K and 80 K Mössbauer spectra was obtained by using four magnetic components: three narrow crystalline sextets of almost identical relative spectral areas (i.e. 15(2)% each) and a broader sextet fitted by a hyperfine field distribution, of 55(2)% relative area (R_A_) contribution and with an average hyperfine magnetic field (< B_hf_ >) of 49.7(2) T at 5 K and 48.4(2) T at 80 K. The hyperfine fields associated to the crystalline sextets are 51.0(1) T, 44.3(1) T and 47.5(1) T at 5 K and 50.1(1) T, 44.0(1) T and 46.7(1) T at 80 K. An increased isomer shift (IS) value (0.98(2) mm/s at 5 K and 0.92(2) mm/s at 80 K) corresponds to the component of the lowest B_hf_ (44.3 T at 5 K) relative to the rest of the components having lower IS (~ 0.6 mm/s at 5 K and 0.5 mm/s at 80 K). Such hyperfine parameters give indications for the following assignment: the crystalline sextets with B_hf_ of 51.0 T and 44.3 T at 5 K correspond to Fe^3+^ and respectively Fe^2+^ ions on octahedral B positions of magnetite, whereas the sextet with B_hf_ of 47.5 T at 5 K corresponds to Fe^3+^ ions on tetrahedral A positions of magnetite. According to R_A_ values, a relatively well crystallized magnetite (with inverse spinel structure) is formed in the core of the nanoparticles which embed almost 45% from the total Fe (in close agreement with the magnetic measurements). The forth sextet with < B_hf_ > of 49.7 T has to correspond to the defected magnetite with disordered magnetic structure in the shell of the nanoparticles, embedding 55% from the total Fe. The Mössbauer spectrum collected at 160 K on sample A200C gives an additional support for the above reasoning of nanoparticles with magnetite cores and magnetic disordered shells. In this case, the best fit was obtained via 3 magnetic components, i.e. two narrower crystalline sextets and a broader sextet which reveals a hyperfine magnetic field distribution with < B_hf_ > of 43.0(2) T. With a corresponding < IS > of 0.5 mm/s and R_A_ of 57(2)%, this last sextet is evidently assigned to the very defected magnetite of disordered magnetic structure in the particle shells. The other two sextets, S1 (R_A_ = 27(2)%, B_hf_ = 44.2(2) T and IS of 0.72(2) mm/s) and S2 (R_A_ = 16(2)%, B_hf_ = 47.8(2) T and IS = 0.34 mm/s) have been assigned to Fe^2.5+^ intermediate valence ions on octahedral B positions and to Fe^3+^ ions on tetrahedral A positions, respectively, as specific to well-formed magnetite above the Verwey transition^37,38^.

The annealing treatment at 300 °C successfully determines the well crystallization of the nanoparticles in the bcc structure of metallic α-Fe, as evidenced by the specific hyperfine parameters at 6 K (*B*_*hf*_ = 34.1(1) T and IS = −0.06(1) mm/s) of the main narrow crystalline sextet (Fig. 9e). However, an additional broader magnetic sextet (B_hf_ = 48.9(5) T) has improved the fit quality, being tentatively assigned to local Fe positions in the bcc structure, randomly surrounded by interstitial C atoms. Such high values of B_hf_ indicate magnetic moments larger than 3µ_B_ and cannot be assigned to an Fe oxide phase due to the corresponding IS of 0.05(3) mm/s specific to a metallic phase.

**Figure 10:**
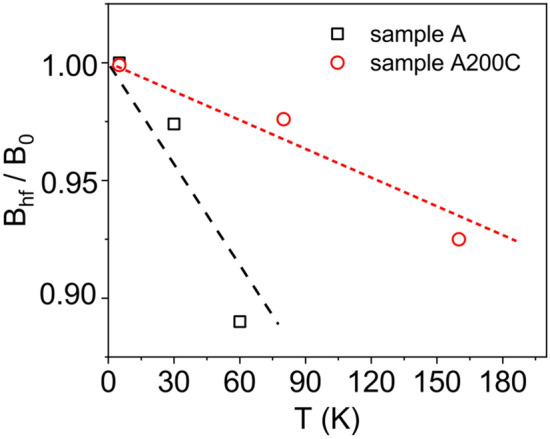
Temperature dependence of the reduced hyperfine magnetic field (B_hf_/B_0_) in samples A and A200C. The dashed lines are linear fits of the experimental data in the regime of collective excitations.

The evolution of the reduced hyperfine magnetic field *B*_*hf*_/*B*_0_ (with *B*_0_ the *B*_*hf*_ value at the lowest temperature of 5 K in this case) for the as-prepared and annealed samples, A and A200C, is shown in Fig. 10. In the case of the as-prepared sample, the average hyperfine magnetic fields provided by the probability distributions were considered. For the A200C sample, the hyperfine magnetic fields of magnetite provided by the weighted average of the *B*_*hf*_ values corresponding to all the discrete sextet contributions were considered. It can be observed that the temperature induced decrease of the *B*_*hf*_/*B*_0_ values for sample A is much faster than for sample A200C, providing evidence of much finer maghemite NPs than of the magnetite NPs.

In the case of non-interacting magnetic NPs relaxing in the regime of collective excitations, the hyperfine magnetic field decreases linearly with the temperature according to the equation^28,33^:

4 $$ B_{hf} = B_{0} \left( {1 - \frac{{k_{B} T}}{2KV}} \right) $$

where, *B*_0_ is the *B*_*hf*_ value in the magnetic frozen regime, e.g. much below *T*_*B*_, *k*_*B*_ is the Boltzmann’s constant, *K* is the anisotropy constant and *V* is the volume of the nanoparticle.

From Eq. (4), the barrier energy *KV* of magnetic NPs in samples A and A200C can be obtained, assuming that the anisotropy constant is an effective anisotropy constant assigned to the overall volume of the core–shell NP. The *KV* values obtained from the experimental slopes of the linear dependences in Fig. 10 are shown in Table 2.

**Table 2:** The calculated energy barrier *KV* of NPs in samples A and A200C.

Sample code	Type of NP	*KV* (10^–20^ J)
A	With γ-Fe_2_O_3_ core	0.34 (2)
A200C	With Fe_3_O_4_ core	1.20 (2)

An absolute value for the effective anisotropy constant of core–shell magnetite NPs with average size of 10 nm in sample A200C of about 2.4 × 10^4^ J/m^3^ is straightforwardly obtained from Table 2. This value is double relative to the typical magneto-crystalline anisotropy of bulk magnetite, indicating rather extrinsic origin due to irregular shape and/or the shell–core magnetic structure. In a similar way, a much higher effective anisotropy constant, i.e. of about 8.2 × 10^4^ J/m^3^, is obtained for the NPs with average size of 5 nm in sample A. This value is reasonably close to the value of 7.7 × 10^4^ J/m^3^ reported by^39^ for NP of average size of 6 nm with maghemite core, providing so an additional support for maghemite core/shell configuration of NPs in the as-prepared sample A.

A final discussion related to the contradictory aspects of the exchange bias fields induced at the lowest measuring temperatures by in zero field and in applied field cooling procedure in samples A and A200C deserves to be mentioned in the context of the different core–shell configurations in the two samples. To note here that such atypical exchange bias structures of type ferrimagnet/spin disordered layer, which deviate from the typical ferromagnetic/antiferromagnetic structures, were previously reported in excellent reviews by Nogues^40,41^ and Phan^42^.

More specifically, at 5 K, an exchange bias field of − 85 Oe is observed in sample A200C relative to a much lower shift of only − 10 Oe for sample A, in the case of the zero field cooling (at remanence) procedure. By contrary, after cooling the samples in only 50 Oe applied field, the exchange bias field increases at − 200 Oe in sample A200C and at an even much higher value of − 440 Oe in sample A. The explanation is related to the value of the net spin induced at the core–shell interface in the magnetically disordered shell side by the ferrimagnetic core structure, which is at remanence in the first case and ordered by the field in the second case. As also proven by MS, a higher spin disorder is expected in the 3.5 nm maghemite core of NPs in sample A relative to the 6.5 nm well-formed magnetite core of the NPs in sample A200C. Therefore, after cooling the samples without applied field (under a higher remanent magnetic polarization of the core in sample A200C), the exchange bias field of this sample has to be higher relative to the one of sample A. However, by the field cooling procedure, the magnetic polarization of the NP cores should be comparable in the two samples and the magnitude of the exchange bias field is imposed only by the representative size of the ferrimagnetic phase in the core. As previously reported in the case of thin film structures^28,30,40^, H_E_ is inversely proportional to the thickness of the ferrimagnetic layer interfaced to the antiferromagnetic one. In the present case, the size of the maghemite core of NPs in sample A is almost half of the size of the magnetite core of NPs in sample A200C and therefore an almost double value of H_E_ would be expected in sample A (experimental values are − 440 Oe relative to − 200 Oe).

## Conclusions

Studies concerning the influence of annealing treatments in hydrogen atmosphere on the local structure and magnetic properties of Fe oxide nanoparticles obtained by laser pyrolysis are presented in this report. The pristine samples were formed by Fe oxide nanoparticles with an average size of about 5 nm and with a core–shell structure consisting of a better formed maghemite core (about 3.5 nm size) and a magnetically disordered shell. The annealing treatment performed at 200 °C in hydrogen atmosphere induces a partial reduction of Fe, giving rise to nanoparticles with an average size of about 10 nm and with a core–shell structure consisting of a very well formed magnetite core (about 6.5 nm size) and a magnetically disordered shell. Annealing treatments at 300 °C in hydrogen atmosphere succeeded to induce the formation of a α-Fe-like phase with the metallic nanoparticles (tens of nm in size) remaining stable with oxidation. In agreement with these changes in phase composition, as well as due to increase in particle size and crystallinity improvement, *T*_*B*_, *H*_*C*_, M_S_ and M_R_ values increase significantly after annealing at 300 °C. As for example, the saturation magnetization of the newly formed metallic phase is more than 50% higher than in bulk metallic Fe. Therefore, hydrogenation treatments seem to be even more effective in this respect as compared to more expensive and time consuming nitriding treatments leading to ordered iron nitride with martensite structure of high saturation magnetization^43^. Specific aspects related to the unidirectional anisotropy of nanoparticles with core–shell magnetic structures assigned to the as prepared sample and the partially reduced sample are also discussed in detail.

## Supplementary information

Supplementary Information.

## Data Availability

The datasets generated during and/or analysed during the current study are not publicly available from the corresponding author on reasonable request.
