# Supplementary material for: Tuning structural and magnetic properties of Fe oxide nanoparticles by specific hydrogenation treatments
Source: Sci Rep. 2020 Oct 14;10:17174. doi: 10.1038/s41598-020-74188-5 (PMC7560822; doi:10.1038/s41598-020-74188-5)
Supplement: Supplementary file 1 — Supplementary Information. [file 41598_2020_74188_MOESM1_ESM.pdf]

Supporting Information for

Tuning structural and magnetic properties of Fe oxide nanoparticles by specific hydrogenation treatments

S.G. Greculeasa[1], P. Palade[1], G. Schinteie[1], A. Leca[1], F. Dumitrache[2], I. Lungu[2], G. Prodan[3], A. Kuncser[1], V. Kuncser[1,*]

[1] *National Institute of Materials Physics, Atomistilor 405A, 077125, Magurele, Romania*

[2] *National Institute for Laser, Plasma and Radiation Physics, 077125, Magurele, Romania*

[3]*Ovidius University of Constanta, 124 Mamaia Avenue, 9005127, Constanta, Romania*

Direct and indirect information supporting the specific core-shell structure of NPs in samples A and A200C.

The unidirectional anisotropy evidenced in samples A and A200C was interpreted in terms of a specific core-shell structure of Fe oxide NPs in these samples. Accordingly, the NPs were assumed to consist in a magnetic core with more ordered ferrimagnetic structure (Ferri) and a surrounding shell of same phase as in the core, but with a more disordered magnetic structure (Diso). Unidirectional anisotropy related effects at Ferri/Diso interfaces (with Ferri replacing the typical ferromagnetic phase and Diso replacing the typical antiferromagnetic phase in classical ferromagnetic/antiferromagnetic structures) have been previously reported. However, the Diso phase in the shell, based on the same Ferri phase as in the core (mainly maghemite in sample A and magnetite in sample A200C), involves an increased number of local structural and/or chemical defects (cation and anion vacancies or substitutional/interstitial impurity atoms). Such local defects may perturb seriously the local magnetic interactions between the Fe cations carrying the magnetic moments, giving rise to a specific magnetic disordered structure/magnetic dead layer. To note that such a specific magnetic structure does not necessarily involve a change of the crystalline structure, which at the opposite limit can go into an amorphous one. In this context, the additional HRTEM images obtained by using a JEOL 2100 electron microscope on NPs of sample A provide only a partial support for a core-shell structure. The high magnification image presented in figure S1

shows several NPs in zone axis orientation with smooth frontiers which seem to be structurally disordered (amorphous), but also NPs preserving the crystalline structure in the entire volume. Because both the defocus effects of the electron optics as well as the expectation for a disordered magnetic structure in the shell which can or cannot be accompanied by structural disorder have to be considered, one may assume that the direct evidence by Electron Microscopy for this specific core-shell structure of NPs is not concluding.

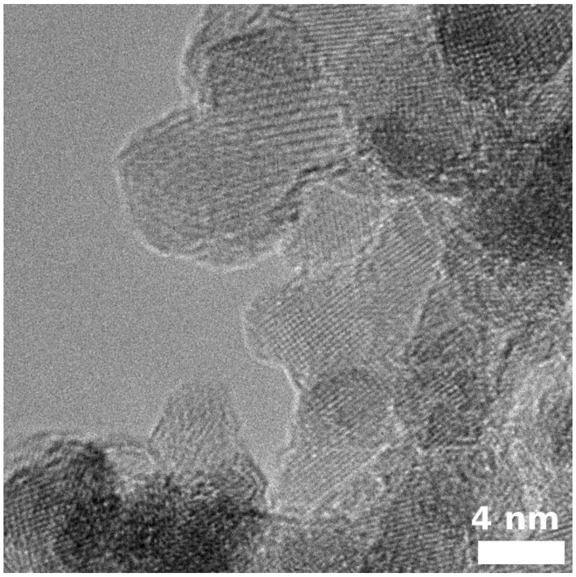

Figure S1 HRTEM image of NPs in sample A. Core-shell-like structures might be tentatively supposed (see central part of the figure) only for those NPs where the magnetic disorder in the shell is accompanied by structural disorder.

However, the magnetic measurements corroborated with the Mössbauer spectroscopy provide, even indirectly, a clear evidence for the above mentioned specific core-shell structure of NPs in samples A and A200C. The fact that all NPs in the sample present similar phase composition (with magnetic ordered core and magnetic disordered shell) are clearly sustained by the temperature dependent Mössbauer spectra showing a unique magnetic relaxation mechanism. If the NPs in the sample would had been formed by two different Fe oxides, two different relaxation mechanisms would have taken place, as reflected by two different collapsing processes of the low temperature Mössbauer sextet.

To note that the real NPs present irregular shapes which are roughly considered as spherical. Expectedly, both the core and the shell present irregular shapes, with the shell

surrounding also irregularly the core (the incomplete surrounding of the core by the disordered shell is also possible). Due to thermodynamic and symmetry reasons, spherical cores and uniform surrounding shells are in average assumed for the constituent NPs in a rough approximation.

Further on, micromagnetic computations have been performed on the above mentioned model of NP, with the geometrical characteristics close to the ones reported for sample A200C, in order to check the possibility of reaching the experimentally obtained values for the coercive field and exchange bias field. The simulations have been performed within the Object Oriented Micromagnetic Framework (free software). A 10 nm diameter sphere has been designed as a core shell-nanoparticle with a 7 nm core with aligned magnetic moments and a 3 nm thick shell with fixed magnetic moments along randomly distributed directions (a slight preference for the Ox orientation in the magnetic disordered shell has been also induced in order to simulate the effect of the field cooling procedure).

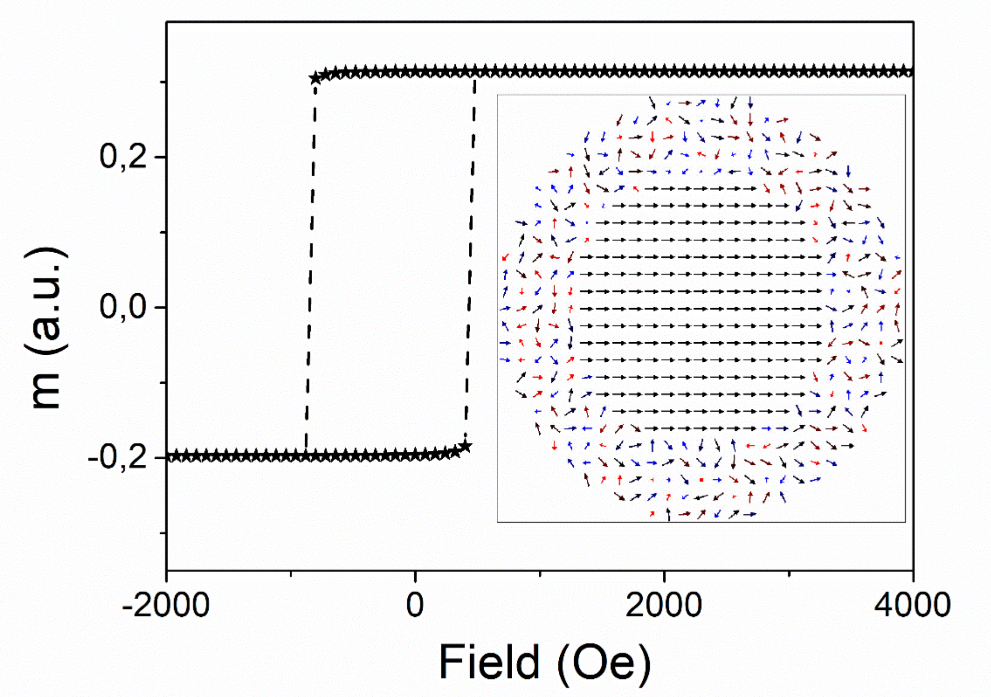

Figure S2 Computed hysteresis loop of a NP with core-shell structure and with a low amount of uncompensated spins in the disordered magnetic structure of the shell. The magnetic parameters mentioned in the text have been considered. The initial magnetic structure of the core-shell nanoparticle in positive saturation field is shown in the inset.

The working space with the initial magnetic structure of the core-shell nanoparticle in positive saturation field is shown in figure S2. The hysteresis loops have been simulated in a magnetic field sweeping a domain from 4000 Oe to -4000 Oe. The elemental cell was 0.4 × 0.4 × 0.4 nm^3 approaching the typical size of the unit cell of magnetite. Values of the spontaneous magnetization specific to magnetite have been assigned to both the core and the shell (M_{core} = M_{shell} = 4.8 × 10^5 A/m) and an uniaxial magnetic anisotropy close to the one derived in this paper for NPs in sample A200C has been considered (K = 2 × 10^4 J/m^3). The exchange stiffness constant in the core was taken as specific to bulk magnetite (A_{core} = 7 × 10^{-12} J/m) whereas the core-shell interaction has been accounted by an exchange stiffness constant of the order of the exchange stiffness constant in the core divided to the square root of interfacial spins in the shell.

The computed hysteresis loop presented in Figure S2 has been calculated for realistic values of the exchange stiffness constant associated to the core-shell interaction, i.e. $A_{core\text{-}shell}$ = 0.02 × 10^{-12} J/m and a net spin induced in the magnetic disordered shell of about 4μ_B per 30 Fe cations, as estimated from the vertical shift of the loop. The values of the coercive field and the exchange bias field are very close to the ones reported for low temperature magnetic measurements on sample A200C (coercive field of about 650 Oe and exchange bias field of about 200 Oe), supporting the validity of the chosen model. The slight vertical shift of the calculated hysteresis loop is due to the amount of uncompensated spins in the shell, pointing toward the positive field and providing a positive contribution to the magnetization, independent to the value of the sweeping field.
